# Impacts of Climate Change on the Global Invasion Potential of the African Clawed Frog *Xenopus laevis*

**DOI:** 10.1371/journal.pone.0154869

**Published:** 2016-06-01

**Authors:** Flora Ihlow, Julien Courant, Jean Secondi, Anthony Herrel, Rui Rebelo, G. John Measey, Francesco Lillo, F. André De Villiers, Solveig Vogt, Charlotte De Busschere, Thierry Backeljau, Dennis Rödder

**Affiliations:** 1 Herpetological Section, Zoologisches Forschungsmuseum Alexander Koenig, Bonn, Germany; 2 UMR7179 CNRS/MNHN, Paris, France; 3 UMR CNRS 5023 LEHNA, University Lyon 1, Lyon, France; 4 UMR CNRS 6554 LETG-LEESA, University of Angers, Angers, France; 5 Centre for Ecology, Evolution and Environmental Changes, Faculdade de Ciências da Universidade de Lisboa, Lisboa, Portugal; 6 Centre for Invasion Biology, Department of Botany & Zoology, Stellenbosch University, Private Bag X1, Matieland 7602, Stellenbosch, South Africa; 7 Dipartimento di Scienze e Tecnologie Biologiche Chimiche e Farmaceutiche, Università di Palermo, Palermo, Italy; 8 Royal Belgian Institute of Natural Sciences, Brussels, Belgium and University of Antwerp, Antwerp, Belgium; University of South Dakota, UNITED STATES

## Abstract

By altering or eliminating delicate ecological relationships, non-indigenous species are considered a major threat to biodiversity, as well as a driver of environmental change. Global climate change affects ecosystems and ecological communities, leading to changes in the phenology, geographic ranges, or population abundance of several species. Thus, predicting the impacts of global climate change on the current and future distribution of invasive species is an important subject in macroecological studies. The African clawed frog (*Xenopus laevis*), native to South Africa, possesses a strong invasion potential and populations have become established in numerous countries across four continents. The global invasion potential of *X*. *laevis* was assessed using correlative species distribution models (SDMs). SDMs were computed based on a comprehensive set of occurrence records covering South Africa, North America, South America and Europe and a set of nine environmental predictors. Models were built using both a maximum entropy model and an ensemble approach integrating eight algorithms. The future occurrence probabilities for *X*. *laevis* were subsequently computed using bioclimatic variables for 2070 following four different IPCC scenarios. Despite minor differences between the statistical approaches, both SDMs predict the future potential distribution of *X*. *laevis*, on a global scale, to decrease across all climate change scenarios. On a continental scale, both SDMs predict decreasing potential distributions in the species’ native range in South Africa, as well as in the invaded areas in North and South America, and in Australia where the species has not been introduced. In contrast, both SDMs predict the potential range size to expand in Europe. Our results suggest that all probability classes will be equally affected by climate change. New regional conditions may promote new invasions or the spread of established invasive populations, especially in France and Great Britain.

## Introduction

Plenty of evidence exists for impacts of climate change on ecosystems and ecological communities [1–8] Climate change modifies climatic factors such as ambient temperatures, precipitation, and the frequency of extreme weather events, which profoundly affects species’ geographical distributions [9]. Recent research reveals coherent patterns of ecological change across systems [10] concerning phenological changes, geographic range shifts and modifications in species abundance [11]. Rising ambient temperatures might promote range expansions beyond the northern range limits or favour altitudinal range shifts, while increasing temperatures enhance winter survival [9].

Non-indigenous species are expanding worldwide [12], and have been identified as a major driver of global biodiversity loss and environmental change [13–16]. Invasive species alter productivity, hydrology and nutrient cycles and thus, influence survival of native species and disrupt natural competition in host ecosystems [17].

Human-mediated transport for tourism or trade provides introduction pathways and therefore contributes to the rising number of introductions of alien species [18, 19]. Increases of biological invasions were found to coincide with the industrial revolution [18, 20, 21] and unprecedented acceleration of merchandise trade within the last 50 years led to progressive increases in the introduction of alien species [18]. Biological invasions are assumed to increase in the future in response to globalisation and climate change [18, 20–23]. Climate change is widely considered to exacerbate the impact of invasive species by making additional space suitable, enhancing survival and reproduction success, and by improving the competitive capacity of non-indigenous species [9, 24–26].

Depending on the physiological sensitivity to climatic conditions, the impact of climate change will vary among organisms (e.g. [27–29]) with poikilothermic taxa, such as amphibians, being particularly affected [11, 28, 30]. Due to behavioural traits, physiological processes and breeding phenology that closely depend on temperature and moisture, and a limited dispersal capacity, amphibians are especially sensitive and will be heavily affected by climate change [11, 31, 32]. However, climate change might also create opportunities for niche differentiation and evolution, by altering the composition of the resident biota, creating empty niches [33]. Further, impacts of climate change will likely vary geographically (e.g. [5, 11, 34, 35]). Although, considerable interest exists in predicting the spread and success of non-indigenous species, research linking climate change and biological invasions remain scarce [9, 36]. While the invasive potential of numerous species such as the invasive cane toad (*Bufo marinus*) in Australia [37, 38] was predicted to increase (e.g. [9, 39–41]) other studies suggest an opposite pattern e.g. for the American Bullfrog (*Lithobates catesbeianus*) in South America [42].

The African clawed frog, *Xenopus laevis* (Daudin, 1802), is one of the world's most widely distributed amphibians with populations originating from the Cape region in South Africa having become established on four continents (North America, South America, Asia and Europe) due to both accidental escape and voluntary release of laboratory animals [43–51]. While the establishment of introduced populations was most successful in areas with a Mediterranean climate, which closely resembles the environmental conditions of the Western Cape region, the persistence of several populations in cooler environments for decades suggests a capacity for long-term adaptation [52]. Recent research indicates that the global invasion potential of *X*. *laevis* has been severely underestimated with vast areas being potentially susceptible to invasion [50]. In addition, it has been claimed that climate change is likely to enhance this species’ invasion potential, favouring range expansion and population growth [53] particularly in Mediterranean climate regions.

Macroecological approaches represent a popular analytical tool to assess and predict the impacts of climate change (e.g. [54–56]) as well as to assess spatial patterns of biological invasions in order to prioritize regions for the early detection of invasion outbreaks (e.g. [42, 57, 58]). The utility of Species Distribution Models (SDMs) to predict future spread of non-indigenous species has been demonstrated repeatedly (e.g. [57, 59, 60]). For general assumptions and methods see Elith and Lethwick [61]. Previous research targeting *X*. *laevis* showed large areas in Asia, southern Australia, south-western Europe, North and South America to be particularly vulnerable to colonization [52]. Owing to its ability to settle on various continents and its expected impact on local wetland communities [46, 49, 62–65], *X*. *laevis* is recognized as a major invasive amphibian species worldwide [50]. For such species it is crucial to develop accurate models of potential colonization and range shifts accounting for short-term climate changes. Such models may help to identify zones that could be colonized, refine risk assessments, and target prevention measures. Aiming to contribute to the future management of *X*. *laevis*, this study investigates the present and forecasts the future potential invasion range of the species on a global scale, based on an updated and extended occurrence data set and a similar set of bioclimatic variables as those used by Measey *et al*. [50].

We hypothesize that rising ambient temperatures associated with climate change will likely increase occurrence probabilities in currently cooler environments e.g. along the present day northern range limits of the species. Thus, climate change will promote the expansion of existing populations and increase the probability of additional invasions in the northern hemisphere, while occurrence probabilities for populations from the southernmost range limits are likely to decrease.

## Materials and Methods

In order to assess the present and future invasion potential of *Xenopus laevis* on a global scale georeferenced occurrence records, covering the species’ native distributional range in South Africa, as well as all known invasive populations in Europe, were obtained from recent literature [50] and supplemented by 286 new records collected during own field research (J.C., J.S., R.R., J.M., F.L., A.dV.). Until recently *X*. *laevis* was considered a species complex with a number of genetically distinct lineages [66]. Measey *et al*. [50] noted that all invasive populations were from the South African ‘Cape’ clade [67, 68] an area including the winter rainfall region and southern coast of South Africa. Since that time, De Busschere *et al*. [69] determined that the French invasion incorporated lineages from throughout the range of *X*. *laevis*. In addition, recent phylogeographic research supports the recognition of *X*. *petersii*, *X*. *victorianus* and *X*. *poweri* as separate species and therefore confines the native range of *X*. *laevis sensu stricto* to southern Africa (South Africa, Lesotho, Swaziland, Namibia, Botswana, Zimbabwe, Mozambique and Malawi) [70]. The occurrence data set for southern Africa used in this study was adjusted accordingly. It is important to note that this is a novel interpretation of the taxonomy and native range of *X*. *laevis* in comparison to that used by Measey *et al*. [50].

To prevent over‐fit and false inflation of model performance through spatially auto‐correlated species records [71–73], the comprehensive dataset of 1382 records was filtered, and clustered locality records were reduced to a single point within a specified Euclidian distance in environmental space using *the spatially rarefy occurrence data tool* for the *ArcGIS SDM toolbox* [74]. The final dataset used to build SDMs contained 826 records for South Africa, 37 for South America, 24 for North America, and 38 for Europe. SDMs built exclusively on occurrence data from the native range of invasive species tend to underestimate the potential invasive distribution, particularly when projecting onto climate change scenarios [75]. Hence, native and invasive occurrence records were pooled [50, 76] for the computation of SDMs. In this way a maximum amount of information on the species’ realized bioclimatic niche was integrated.

As environmental predictors, 19 bioclimatic variables available through the WorldClim-database ([77] http://www.worldclim.org/bioclim) were used. They represent minima, maxima and average values of monthly, quarterly, and annual ambient temperature as well as precipitation recorded between 1950 and 2000. All predictors had a spatial resolution of 2.5 arc min (approx. ~5 km resolution at the equator). Out of the total set of variables a set of nine predictors with pairwise Spearman rank correlation coefficients R^2^ < 0.75 were selected to minimize predictor correlation.

Subsequently, SDMs were computed using the machine learning algorithm *Maxent* version 3.3.3k [78, 79]. *Maxent* is supposed to exhibit a higher predictive performance than more conventional techniques [61] and has successfully been used to model the potential distribution of invasive species and to assess impacts of climate change [78]. Only linear, quadratic and product features were allowed in order to restrict model complexity, while extrapolation was not allowed to reduce uncertainties due to projections onto non-analogous climates [80, 81]. The *Maxent* model was trained by randomly splitting the species records into 70% used for model training and 30% for model testing applying a bootstrap approach. The Area Under the receiver operating characteristic Curve (AUC) [82] was used to evaluate the discrimination ability of the resulting SDM. Averages across 100 replicates were used for further processing. As the selection of an appropriate background is known to affect model performance [83], a circular buffer of 250 km around each locality record was selected as training area following Measey *et al*. [50].

Ensemble SDMs were computed using the *biomod2* package version 3.2.2 [84] for *Cran R* [85] including the following eight algorithms: Generalized Linear Models (GLM), Generalized Boosting Models (GBM), Generalized Additive Models (GAM), Classification Tree Analysis (CTA), Artificial Neural Networks (ANN), Factorial Discriminant Analysis (FDA), Maxent, and Multivariate Adaptive Regression Splines (MARS) applying the same training background as for the *Maxent* analyses. We applied a bootstrapping approach with 100 replicates randomly subdividing the locality dataset in 70% for model training, whereas the remaining 30% were used for model evaluation using the AUC [82], True Skill Statistic (TSS) and Cohen’s Kappa [86]. The average projection across all replicates was used for further processing. The “Minimum training presence” (mtp) referring to the lowest generated probability estimate of the training data [87], was applied as presence-absence threshold. While threshold selection is a potential source for biases, the mtp has been shown to represent a confident method performing well for presence only SDMs [88] particularly for modelling potential distributions of invasive species [88, 89].

Areas requiring extrapolation beyond the training range of the variables were quantified using a *multivariate environmental similarity surface* (MESS) analysis [90] for *Maxent* and conceptually equivalent clamping masks for *biomod2*.

To predict the future potential distribution of *X*. *laevis* on a global scale, 11 general circulation models (GCMs: BCC-CSM1-1, CCSM4, GISS-E2-R, HadGEM2-AO, hadGEM2-ES, IPSL-CM5A-LR, MIROC-ESM-CHEM, MIROC-ESM, MIROC5, MRI-CGCM3 and NorESM1-M) representing simulations for four representative concentration pathways (RCP2.6, RCP4.5, RCP6, RCP8.5) for 2070 were obtained from the fifth assessment of the Intergovernmental Panel for Climate Change (IPCC AR5 WG1 2013; http://www.ipcc.ch, [91]). The selected RCPs represent four possible greenhouse gas emission trajectories ranging from low (RCP2.6) to high (RCP8.5) corresponding to increases in global radiative forcing, from pre-industrial times to 2100. These climate projections were statistically downscaled to match the bioclim variables using the delta method [77], (http://www.worldclim.org/downscaling) for details also see [92,93]. As differences between the selected GCMs might cause uncertainty in SDM projections [59], average values across all GCMs were calculated for each RCP respectively. Finally, SDMs were projected onto the derived future climate change scenarios.

In order to quantify impacts of different RCP scenarios onto the global invasion potential of *X*. *laevis*, the following predicted areas were determined: a) the entire SDM area using the ‘minimum training presence’ as threshold, b) the respective MESS area, and c) the SDM area–MESS area. A probability cut-off of 25%, 50%, and 75% onto the ‘SDM area–MESS area’ was applied to assess impacts for different probability classes. Further, the full model was partitioned into estimates for each continent. Subsequently, the invasion potential for *X*. *laevis* was determined on a continental scale as described above. Shift maps were generated to illustrate predicted gains, losses and stability of environmentally suitable space for all climate change scenarios following Bertelsmeier *et al*. [94]. Comparisons between the results obtained by our *Maxent* analyses, the results presented by Measey *et al*. [50] and the results obtained via *biomod2* were performed by rescaling the probability output and subtracting the potential distribution grids from each other. Rescaling involved subtracting the minimum training presence threshold from each model and computation of percentages per grid cell relative to the maximum probability. The resulting maps quantitatively indicate for each area which SDM shows a higher probability.

## Results

Model performance was 0.841 (AUC_test_) and 0.846 (AUC_training_) for the maximum entropy model while weighted means of 0.631 for Cohen’s Kappa, 0.892 for AUC, and 0.685 for TSS were obtained for the ensemble model, demonstrating that both SDMs discriminate moderately well between suitable versus unsuitable space [79]. For the maximum entropy model the contribution of eight predictors exceeded 5%, while for the ensemble approach seven predictors had a contribution exceeding 5% (Table 1, S1 Table). Predictor contribution for the maximum entropy model was particularly high for ‘precipitation of the driest quarter’ (27.65%), ‘mean temperature of the wettest quarter’ (16.82%), ‘mean temperature of the coldest quarter’ (14.52%), and ‘precipitation of the warmest quarter’ (11.38%) (Table 1). Variables with high contribution in the ensemble model were ‘mean temperature of the coldest quarter’ (19.05%), ‘precipitation of the warmest quarter’ (16.57%), ‘mean temperature of the warmest quarter’ (13.92%), ‘precipitation of the driest quarter’ (12.56%) (Table 1). For the respective response curves see S1 Fig & S2 Fig.

**Table 1:** Variable contribution for the maximum entropy and the ensemble SDM.

		Variable Contribution (%)	
ID	Bioclimatic Variable	Maxent SDM	Ensemble
Bio 17	precipitation of driest quarter	27.65	12.56
Bio 8	mean temperature of the wettest quarter	16.82	7.61
Bio 11	mean temperature of coldest quarter	14.52	19.05
Bio 18	precipitation of warmest quarter	11.38	16.57
Bio 19	precipitation of coldest quarter	8.25	8.25
Bio 7	temperature annual range	6.99	4.96
Bio 9	mean temperature of driest quarter	6.24	8.33
Bio 16	precipitation of wettest quarter	6.21	7.95
Bio 10	mean temperature of warmest quarter	1.93	13.92

**Fig 1:**
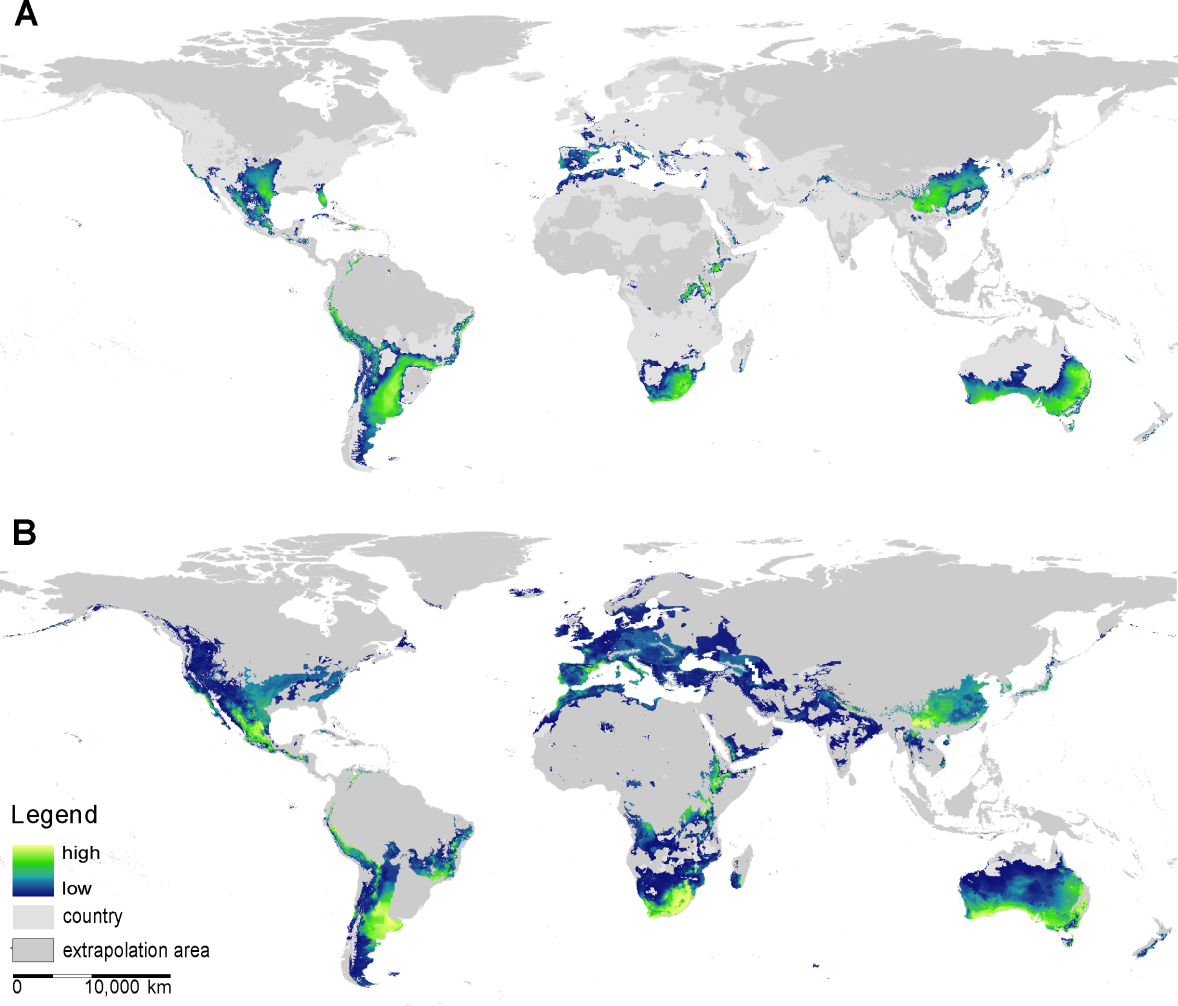
Global projection of the potential distribution of *X*. *laevis* **A)** derived from the maximum entropy SDM; **B)** derived from the ensemble SDM. Probability ranging from moderate (dark blue) to highly suitable (yellow).

Both modelling approaches yielded similar global patterns for the present potential distribution of *X*. *laevis*. However, the more complex ensemble SDM predicted larger areas with slightly higher probabilities than the maximum entropy SDM (Fig 1 & S3 Fig). While the ensemble SDM predicted 28% of the world’s surface to be presently environmentally suitable for *X*. *laevis*, the maximum entropy SDM predicted only 12% (Table 2). As expected, high occurrence probability was predominantly predicted for areas resembling climatic characteristics of the South African Cape region with high annual variation in ambient temperatures, comparatively warm and dry summers, and mild and wet winters (S1 Fig & S2 Fig). More precisely, probability was correlated with mild winter temperatures (10–20°C), low precipitation during the wettest (<350 mm), and the coldest quarter (<500 mm) and high precipitation during the warmest quarter (400–600 mm) (S1 Fig & S2 Fig).

**Table 2:** Environmentally suitable space given as percent of the world’s surface area for current climatic conditions and projections onto climate change scenarios. Percentages refer to the SDM-MESS area; values increasing with climate change scenarios are displayed in bold.

	Maximum entropy SDM				
Continent	% current	% RCP 2.5	% RCP 4.5	% RCP 6	% RCP 8.5
Africa	9	6	5	5	3
Europe	4	**8**	**9**	**10**	**11**
North America	10	8	7	7	7
South America	26	23	21	21	20
Australia	40	33	31	30	28
Asia	8	9	**10**	**10**	**11**
global	12	12	11	11	11
	Ensemble SDM				
	% current	% RCP 2.5	% RCP 4.5	% RCP 6	% RCP 8.5
Africa	24	16	12	12	9
Europe	21	**30**	**33**	**35**	**38**
North America	24	**29**	**31**	**30**	**30**
South America	30	**31**	28	28	26
Australia	74	72	66	66	56
Asia	24	20	19	19	18
global	28	27	26	26	25

According to both models, there are regions exhibiting high occurrence probabilities under current climatic conditions located on all continents (Fig 1), but percentages of environmentally suitable space varied greatly (Table 2). Both SDMs predict only moderate coverage with environmentally suitable space for *X*. *laevis* to presently exist in the northern hemisphere, while high coverage was predicted for Australia and South America (Table 2). Potentially highly suitable areas cover the south central United States (Texas, Kansas), western (stretching from Peru through Colombia and into Venezuela), eastern (eastern Brazil, stretching along the Paraná River), and southern South America (north-eastern Argentina). In Europe, particularly high probabilities are predicted in Portugal, eastern Spain, southern France, and Italy. In accordance with Measey *et al*. [50], both SDM approaches predict only moderate occurrence probability in Great Britain. In addition to the native distribution of *X*. *laevis* covering vast areas in southern Africa, there is a high occurrence probability in Morocco and the eastern Afromontane region (where no invasions by *X*. *laevis* have been reported so far). Moreover, both SDMs predict high suitability in China (Nanzhao plateau, Sichuan, Chinese plain), Japan, and southern Australia. The maximum entropy SDM highlights additional regions with high probabilities in Florida and south-eastern China (Zhejiang Province) (Fig 1, S3 Fig).

**Fig 2:**
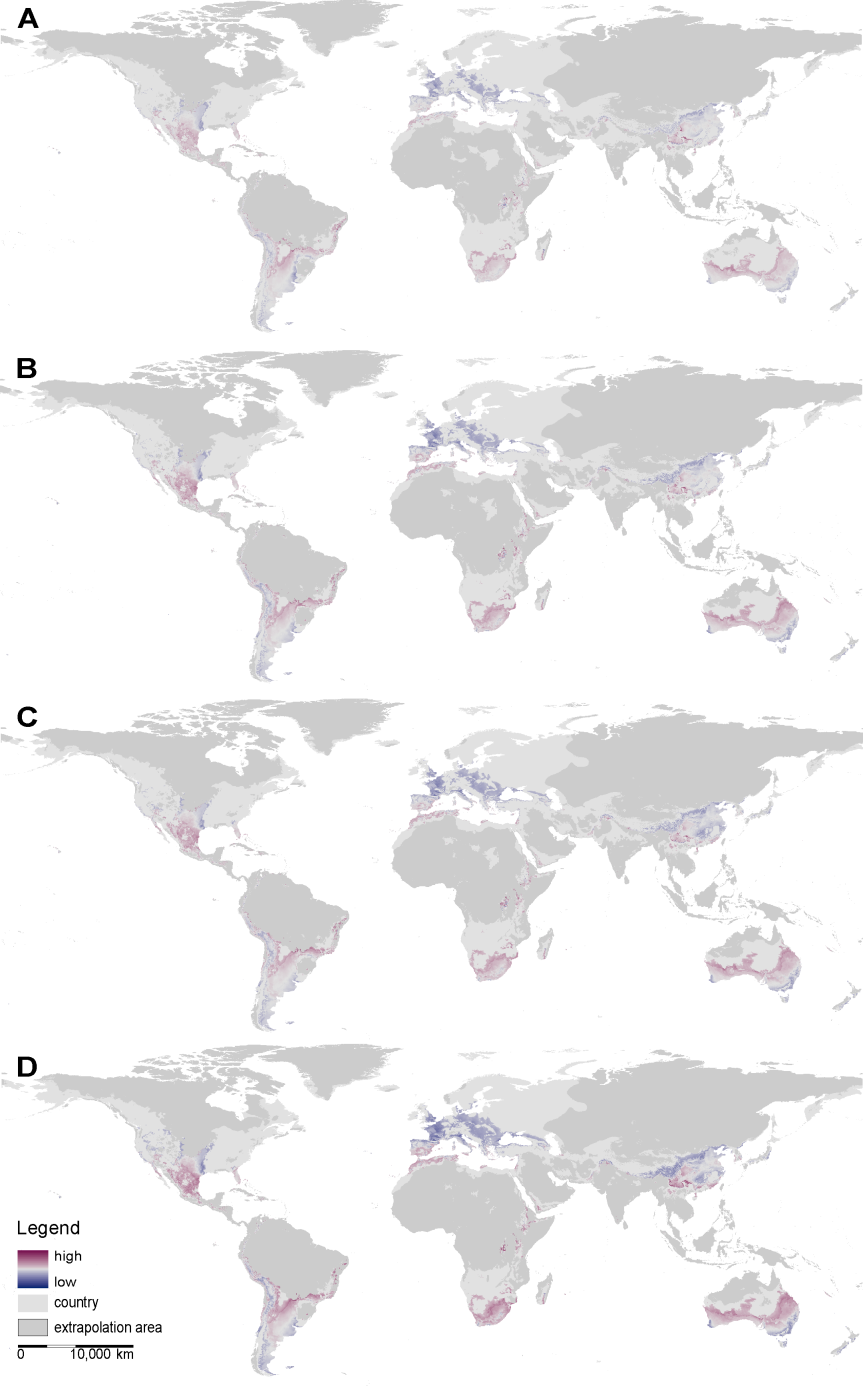
Global shift maps derived from Maxent illustrating predicted gains (dark violet) and losses (dark blue) of environmentally suitable space for different climate change scenarios; **A)** IPCC RCP2.6, **B)** IPCC RCP4.5, **C)** IPCC RCP6, **D)** IPCC RCP8.5.

**Fig 3:**
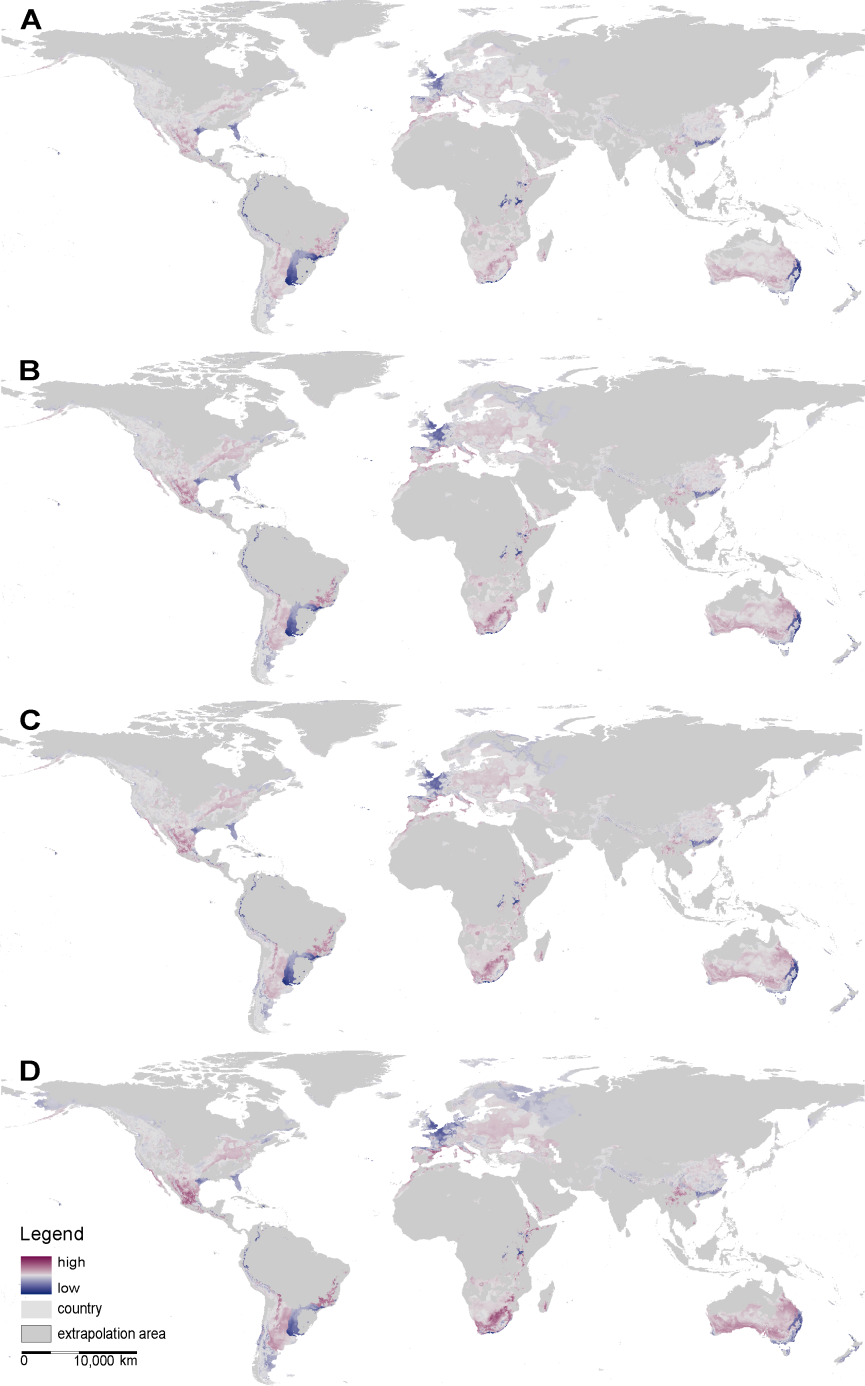
Global shift maps derived from the ensemble SDM illustrating predicted gains (dark violet) and losses (dark blue) of environmentally suitable space for different climate change scenarios; **A)** IPCC RCP2.6, **B)** IPCC RCP4.5, **C)** IPCC RCP6, **D)** IPCC RCP8.5.

**Fig 4:**
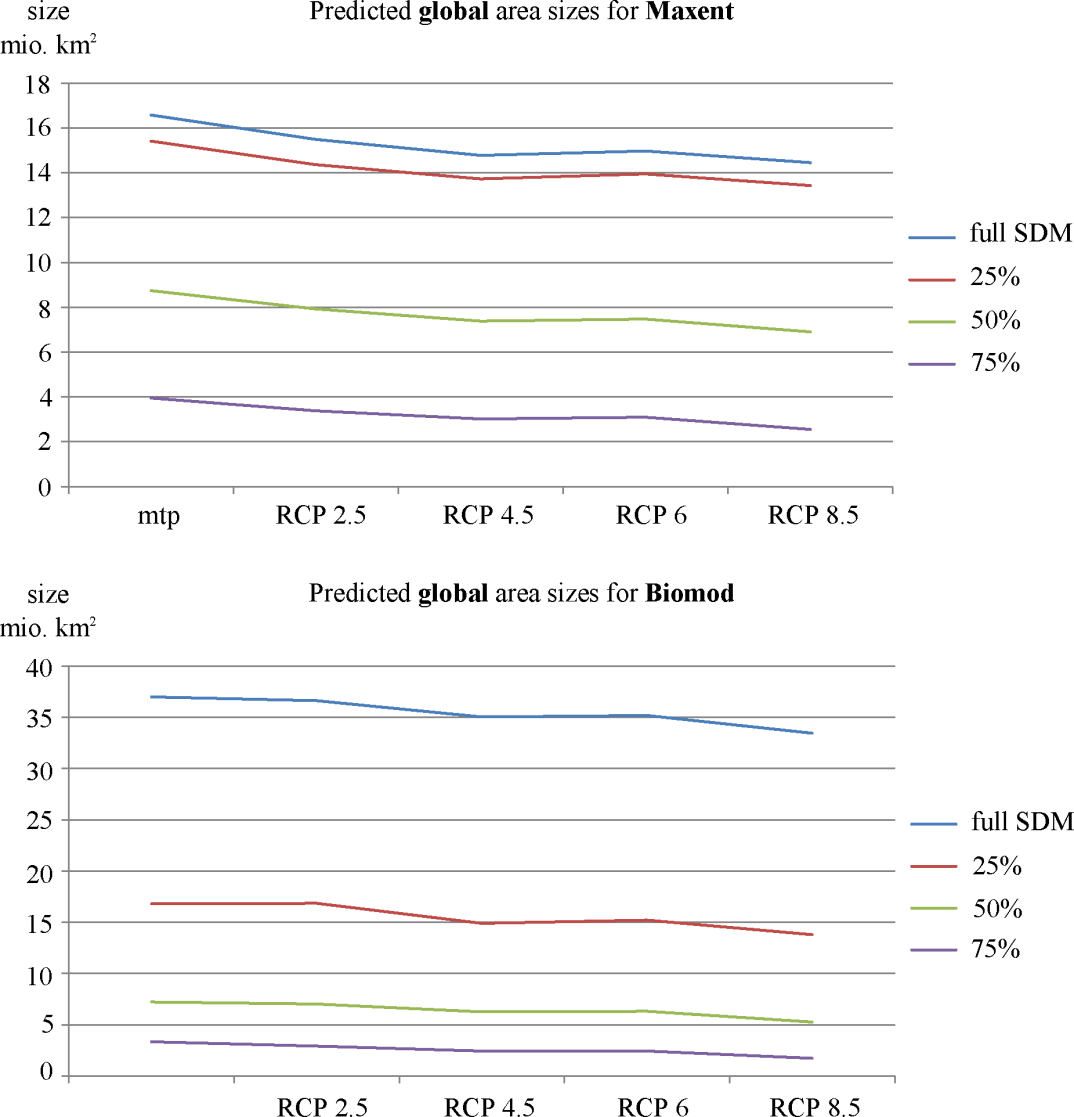
Predicted development of area sizes suitable for *X*. *laevis* on a global scale; a) for the Maximum entropy SDM and; b) for the ensemble SDM. Mtp = minimum training presence, all areas sizes refer to SDM area–MESS area.

On a global scale, both SDM approaches predict suitable range sizes for *X*. *laevis* to decrease across all four RCP scenarios (Table 2, Fig 2 & Fig 3). However, the magnitude of decrease varies between RCP scenarios and between SDM approaches. For the maximum entropy SDM, the potentially suitable range size is predicted to decrease by 7 to 13% from RCP 2.5 to RCP 8.5, respectively. This corresponds to a maximum decrease of 1% in relation to the world’s surface area (Table 2). The ensemble SDM predicts a rather moderate decrease of 1 to 10%. Since the areas predicted by the ensemble SDM are generally larger, the portion of the world’s surface area predicted to be suitable shrinks by 3% (Table 2). A comparison between the full model and the respective probability cut-offs does not reveal significant differences between RCP scenarios (Fig 4), suggesting that probability classes will be equally affected by climate change.

**Fig 5:**
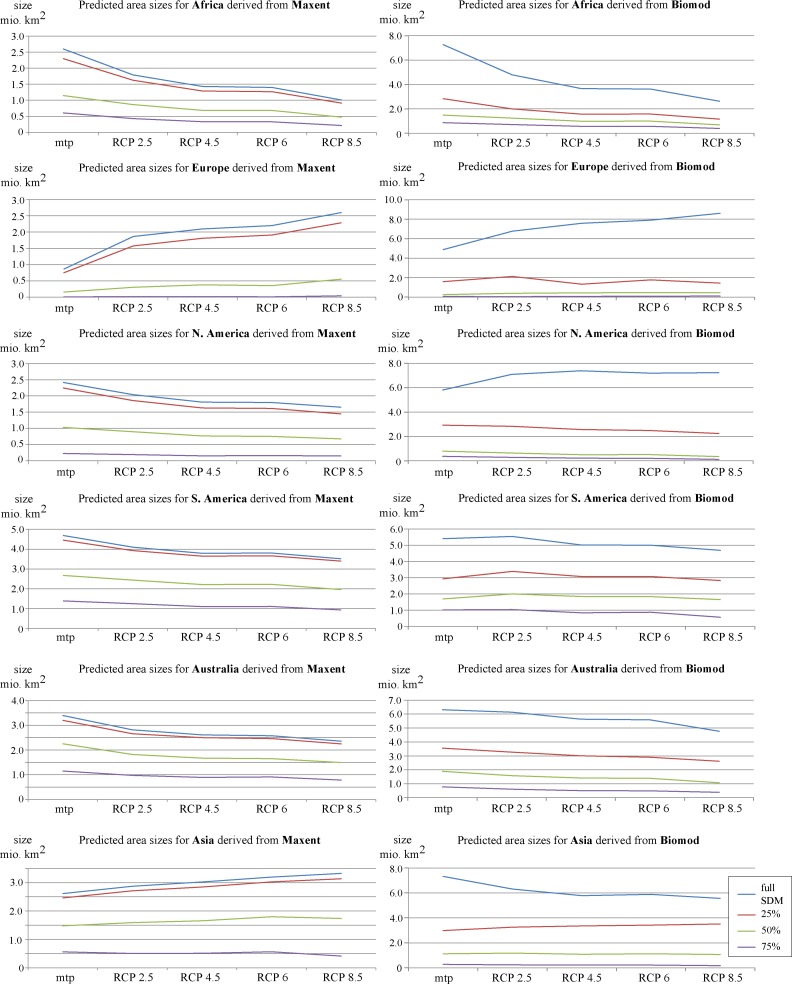
Predicted development of area sizes suitable for *X*. *laevis* on a continental scale; left) for the Maximum entropy SDM, right) for the ensemble SDM. Mtp = minimum training presence, all areas sizes refer to SDM area–MESS area.

On a continental scale, both SDM approaches suggest range sizes to decrease for the species’ native range in South Africa, as well as for the invaded areas in South America and Australia, where the species has not been introduced (Fig 5, Table 2, S2 Table & S3 Table). In contrast, the potential range in Europe will expand in response to climate change (Table 2). Concordantly, shift maps highlight different magnitudes of expected gains in Europe (Fig 2 & Fig 3).

However, there are minor differences between the results of both approaches: while the maximum entropy SDM predicts range sizes to decrease for all probability classes, the ensemble SDM suggests range sizes to increase for North America when applying the mtp threshold (Fig 5). Furthermore, the ensemble SDM predicts increasing range sizes for the >25% probability class in Asia (Fig 5), while the maximum entropy model predicts increases for the mtp threshold, as well as for the probability classes >25% and >50% (Fig 5). Finally, the ensemble SDM predicts increasing range sizes only for the mtp threshold, while the maximum entropy SDM suggests an area expansion across all probability classes in Europe (Fig 5, S2 Table & S3 Table).

## Discussion

Our predictions of the present potential distribution of *Xenopus laevis* generally agree with the findings presented by Measey *et al*. [50] highlighting analogous spatial extents and geographic locations. Both SDMs reveal large areas that can potentially be colonized on several continents. However, the overlap comparison of our SDMs with the prediction by Measey *et al*. [50] yields higher probability values on all continents for both our ensemble and our maximum entropy SDM (S3 Fig). These differences might be attributed to the different occurrence data sets used. While Measey *et al*. [50] applied a restricted definition within *X*. *laevis* using only records from a single clade occurring in the winter rainfall zone and southern coast of South Africa [66], the data used in this study was adjusted according to the findings of Furman *et al*. [70] resulting in a larger coverage for the species native range. Thus, the environmental space covered by the occurrence dataset used was larger than in Measey *et al*. [50].

Under current climatic conditions, both the *Maxent* and the ensemble model identify regions with suitable climatic conditions favouring invasion in Portugal, France, Sicily, California, Chile, and Japan, where invasive populations already exist. Both SDMs predict only moderate probability for Great Britain, where populations from Wales and Lincolnshire have recently been extirpated [53]. Furthermore, our results highlight areas in Spain (including the Balearic Islands), mainland Italy (including Sardinia), and southern France (including Corsica) to be highly vulnerable to potential invasions, as these regions exhibit suitable climatic conditions for *X*. *laevis* and are adjacent to established invasive populations. Globally, the same applies to Baja California and central Mexico.

Future projections of both SDM approaches identify regions that will likely become vulnerable to colonization in response to climate change. With an expected decrease of 1–3% (given as percentage of the worlds’ surface area) the overall magnitude of expected changes appears to be moderate, while the predicted global area suitable for *X*. *laevis* remains stable or slightly decreases with increasing RCP scenarios. However, predictions for Europe are the major exception to this general trend with particularly good prospects for the invasive populations in north-western Europe (Figs 2 & 3). *Xenopus laevis* is capable of enduring extreme conditions ([50] and references therein). However, reproduction seems to be triggered by rainfall and increasing temperatures [95, 96]. These physiological restrictions are well reflected in the variable contributions of the models. These were highest in the precipitation of driest and warmest quarter and in the temperature of the wettest, coldest and warmest quarters affecting reproduction cycles. While reproduction occurs throughout the year in California [97] the lower lethal limit of temperature tolerance in embryos was reported to be 10°C [95]. Harsh conditions only permitted infrequent reproduction in Great Britain [53] and rising temperatures will likely improve physiological performances, fecundity, breeding success, and increase the rate of larval development.

Thus, regional patterns may facilitate new invasions or promote a spread of the established invasive populations, especially in France and Great Britain, where populations persisted for decades [52]. While invasive populations are already spreading in France, British populations are presently considered extirpated [53]. Due to an increased environmental suitability caused by climate change along the northernmost boundaries of the species’ range, chances of successful establishment in Great Britain in case of re-introductions will increase in the future. Climate change is widely considered to exacerbate the impact of invasive species [9, 25] enhancing the invasive potential of some species (e.g. [9, 39–41]), including the invasive cane toad (*Rhinella marina*) in Australia [37, 38]. However, some studies suggest an opposite pattern e.g. for the global invasion potential of an assemblage of ant species [98], or the American Bullfrog (*Lithobates catesbeianus*) in South America [42].

For *X*. *laevis*, it has been claimed that climate change will likely favour range expansion and population growth [53] particularly in Mediterranean climate regions. While on a global scale our predictions reveal the species’ potential range to decrease in response to climate change, populations from the northern hemisphere are predicted to expand.

As *X*. *laevis* is kept as a model organism in laboratories all across the world and is still traded intensively [51, 98] Measey *et al*. [51] emphasize the importance of biosecurity at breeding facilities to prevent further escape and voluntary release of frogs and tadpoles [99]. While by now scientists working with *X*. *laevis* are likely to be aware of the species’ invasion potential [100], this frog is also readily available via the pet trade [98]. Once introduced, the species rapidly disperses using irrigation canals, ponds, and rivers as migration corridors, but also performs terrestrial migrations [101] even without rainfall [102]. Estimated annual spread of feral populations varied between 1 km [101] in France and 5.4 km [62] in Chile. Recent research found the maximum overland dispersal in native populations to be 2.3 km (Euclidean distance) within 6 weeks [102].

Although invasive *X*. *laevis* have demonstrably negative impacts on resident amphibian and fish communities [49, 63, 64, 103], attempts to eliminate invasive populations are limited. Recent studies stressed the urgent need for rigorous and comprehensive invasive species risk assessments to contribute to the development of management strategies [104, 105]. Prevention is generally considered more effective and cheaper than control and eradication of established populations [8, 25]. SDMs represent a quick and cost efficient tool to evaluate the current and future invasion potential of non-indigenous species. In addition, SDMs facilitate the identification of areas with high susceptibility to invasion and help to prioritize management actions. According to our results preventive measures should predominantly focus on the species’ northern range limits, particularly north-western Europe to prevent further spread and establishment of new populations as well as a re-introduction in Great Britain.

Even though considered particularly difficult in Mediterranean areas, eradication of invasive populations of *X*. *laevis* has been proposed [101] and an eradication program was established by the Portuguese Governmental Nature Conservation Institute in Oeiras, western Portugal in 2010 [50]. In addition, eradication was successfully executed in Scunthorpe, Humberside area, Great Britain [50]. According to our results eradication of established populations of *X*. *laevis* should focus on areas where populations are still small and scattered, but likely to expand in response to climate change e.g. Portugal and Sicily.

## Supporting Information

S1 Fig Maximum entropy SDM response curves for selected predictor variables displaying relationships between predictor variables and occurrence probability of *Xenopus laevis*. Model contribution was assessed by building the model using a single corresponding predictor variable. The logistic output (probability of presence) is displayed on the Y axis. Red curves refer to mean responses of 100 replicate Maxent runs while the mean +/- one standard deviation is displayed in blue.
(PDF)

S2 Fig Response curves for ensemble SDM showing the relationships between environmental predictors and occurrence probability of *Xenopus laevis*. The logistic output (probability of occurrence) is displayed on the Y axis.
(PDF)

S3 Fig **A)** Overlap analysis of the ensemble SDM and the maximum entropy SDM, with red highlighting areas where the ensemble SDM yields higher probabilities and blue depicting areas where the maximum entropy SDM yields higher probabilities; **B)** Overlap analysis of the ensemble SDM and the SDM by Measey *et al*. (2012), with red indicating higher probability of the ensemble SDM and blue showing higher values for the SDM by Measey *et al*. (2012); **C)** Overlap analysis of the maximum entropy SDM and the SDM by Measey *et al*. (2012), with red highlighting regions with higher probability of the ensemble SDM and blue showing higher probability values for the SDM by Measey *et al*. (2012). Colour saturation increases with deviation of the models. Areas where both SDMs yield similar probability values are displayed in white.
(PDF)

S1 Table Variable contribution for single algorithms of the ensemble SDM. (XLSX)

S2 Table Predicted area sizes in mio. km² for the maximum entropy SDM; maximum values for each threshold (mtp, 25%, 50%, 75%) are displayed in bold. (XLSX)

S3 Table Predicted area sizes in mio. km² for the ensemble SDM; maximum values for each threshold (mtp, 25%, 50%, 75%) are displayed in bold. (XLSX)
