# Supplementary figures and images for: Impacts of Climate Change on the Global Invasion Potential of the African Clawed Frog Xenopus laevis
Source: PLoS One. 2016 Jun 1;11(6):e0154869. doi: 10.1371/journal.pone.0154869 (PMC4889038; doi:10.1371/journal.pone.0154869)

bio10

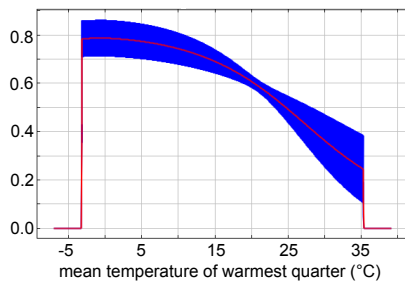

bio11

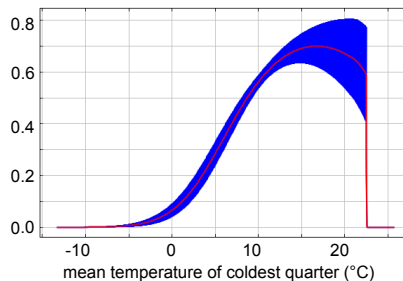

bio16

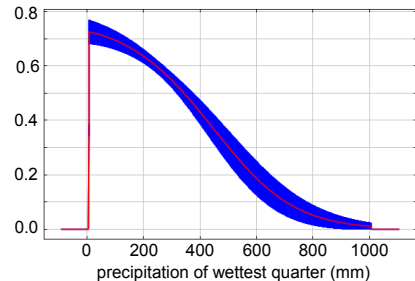

bio17

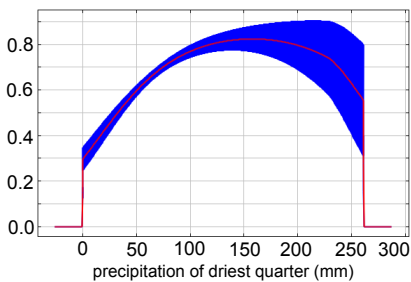

bio18

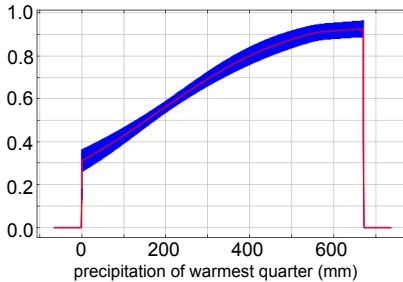

bio19

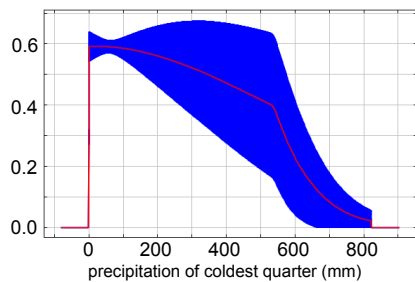

bio7

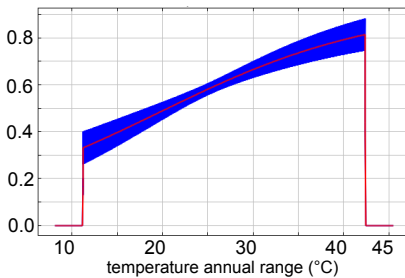

bio8

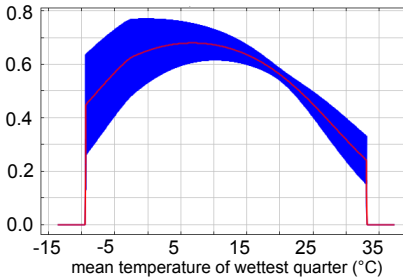

bio9

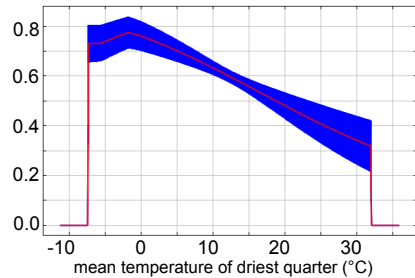

Supplement: S1 Fig — Model contribution was assessed by building the model using a single corresponding predictor variable. The logistic output (probability of presence) is displayed on the Y axis. Red curves refer to mean responses of 100 replicate Maxent runs while the mean +/- one standard deviation is displayed in blue. (PDF) [file pone.0154869.s001.pdf]

bio10

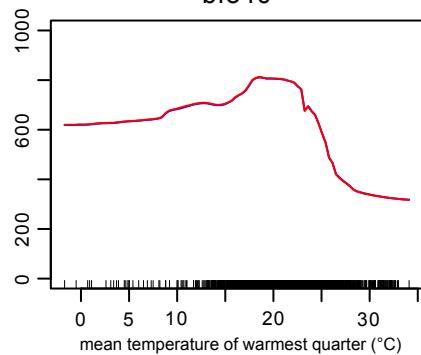

bio11

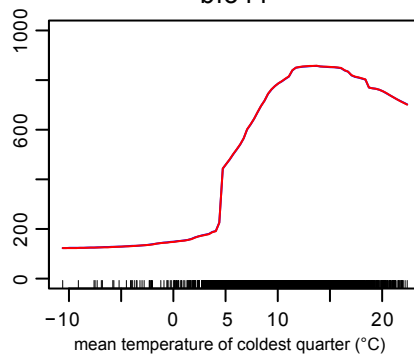

bio16

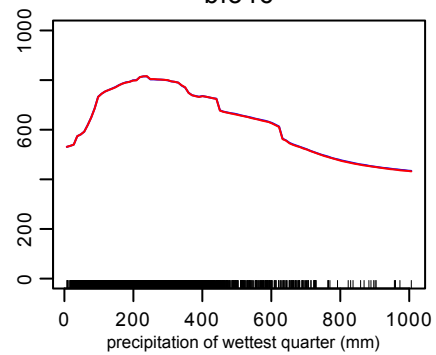

bio17

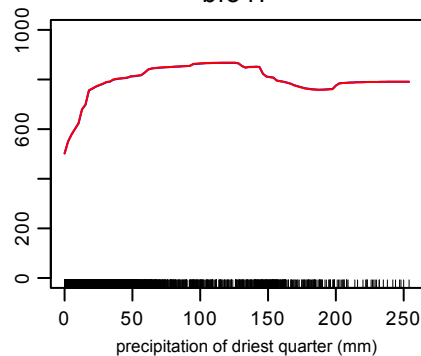

bio18

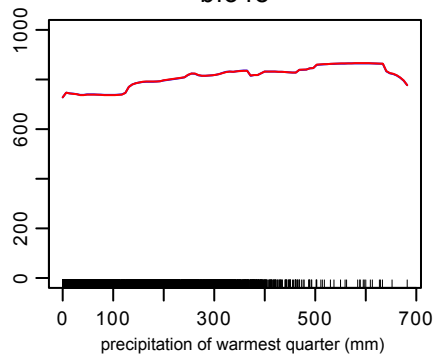

bio19

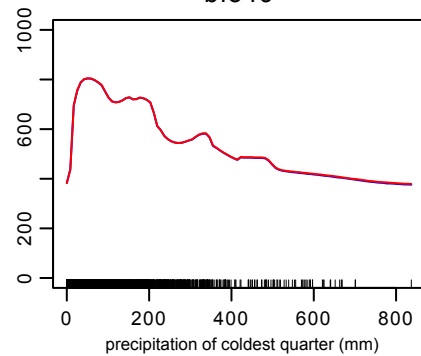

bio7

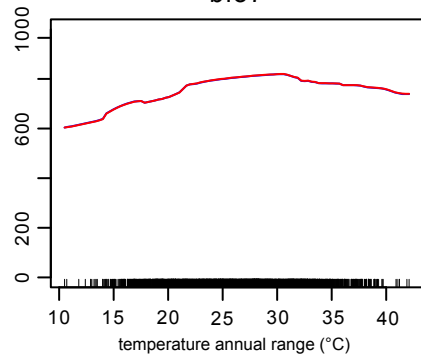

bio8

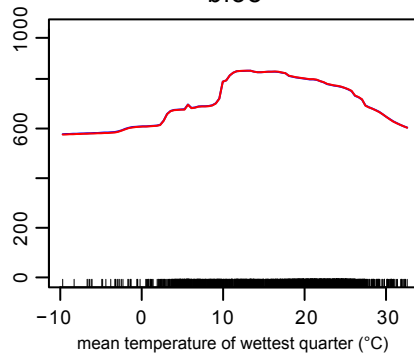

bio9

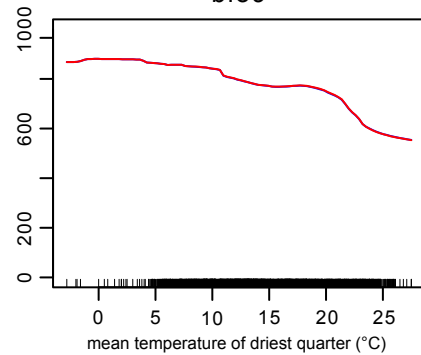

Supplement: S2 Fig — The logistic output (probability of occurrence) is displayed on the Y axis. (PDF) [file pone.0154869.s002.pdf]

**A**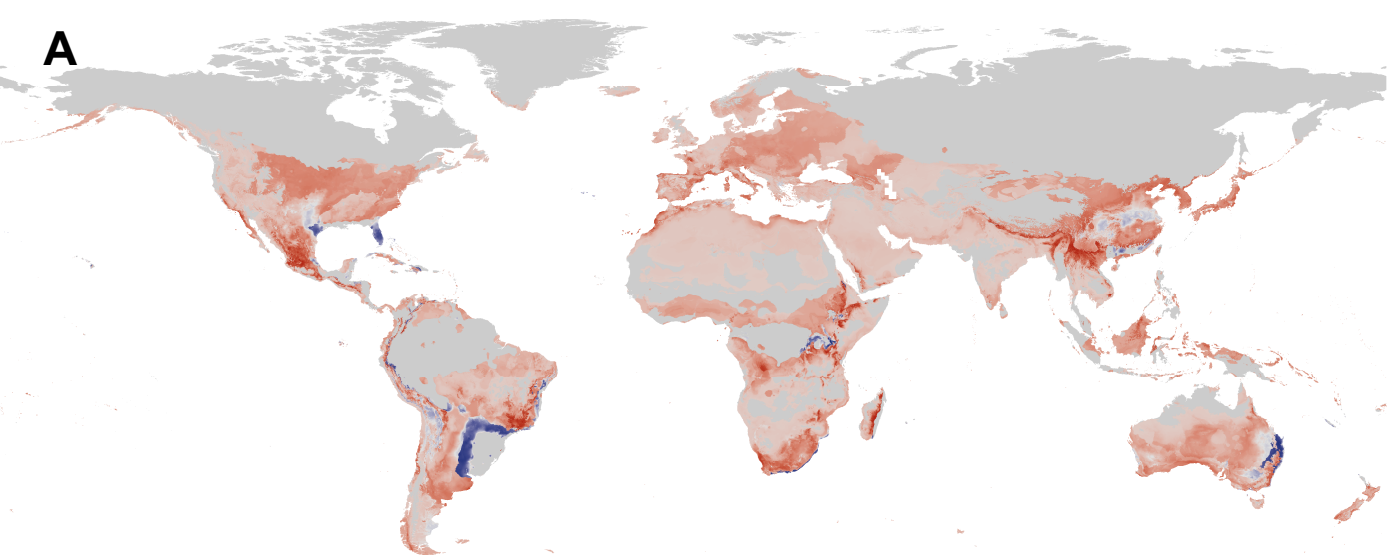**B**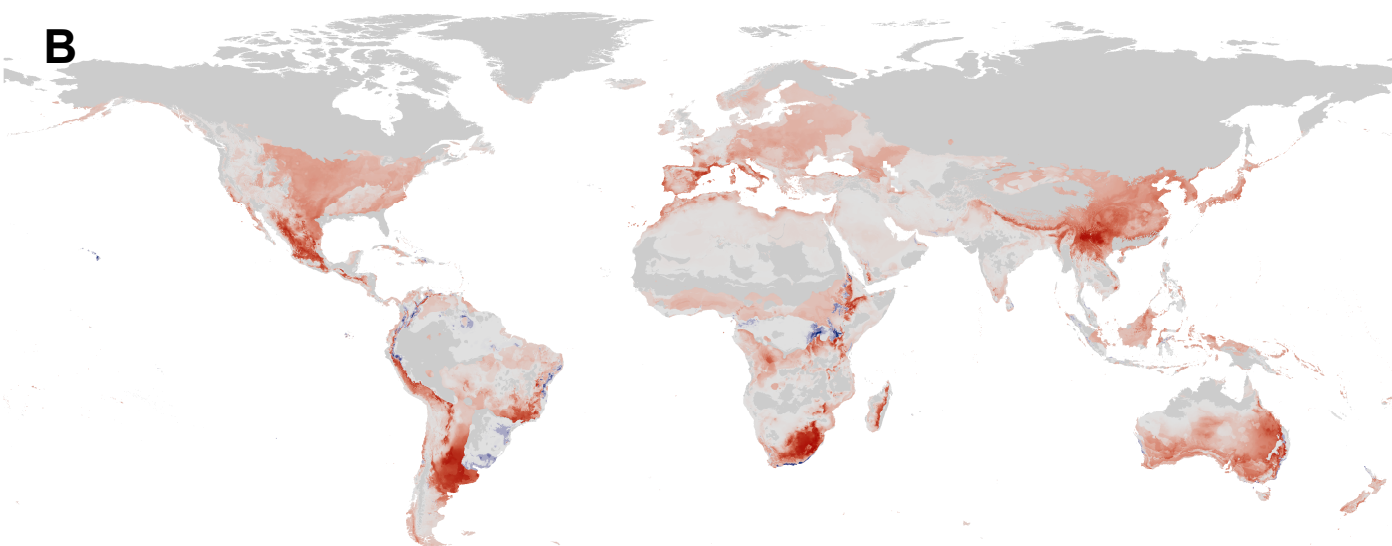**C**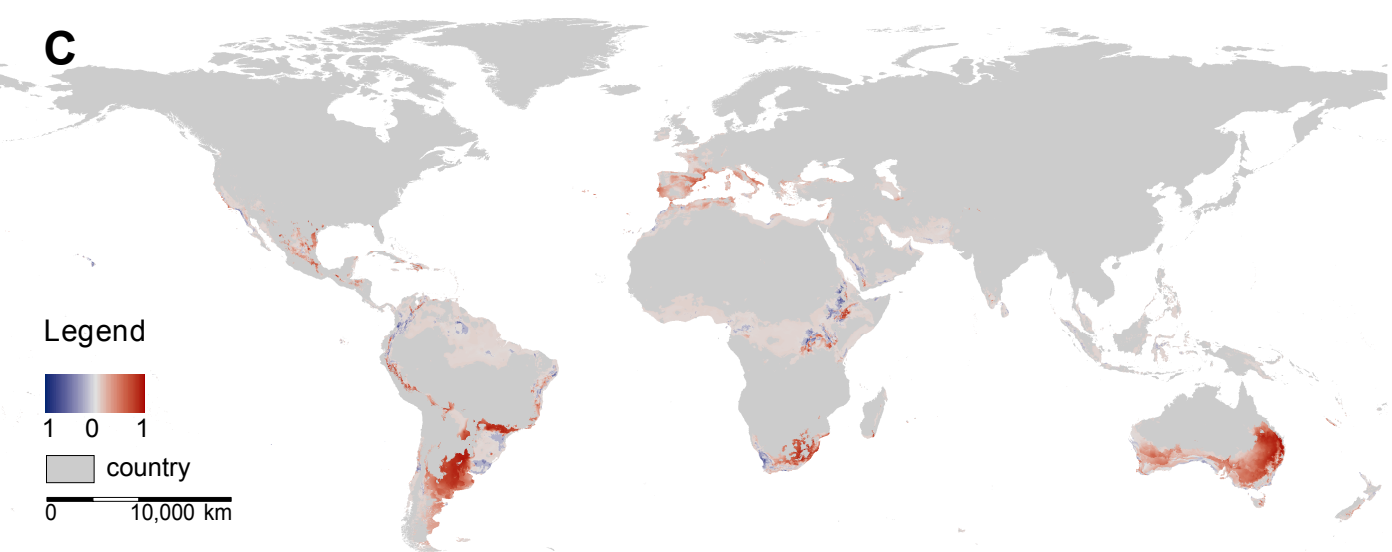**Legend**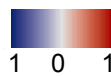

1 0 1

country

0 10,000 km

Supplement: S3 Fig — A) Overlap analysis of the ensemble SDM and the maximum entropy SDM, with red highlighting areas where the ensemble SDM yields higher probabilities and blue depicting areas where the maximum entropy SDM yields higher probabilities; B) Overlap analysis of the ensemble SDM and the SDM by Measey et al. (2012), with red indicating higher probability of the ensemble SDM and blue showing higher values for the SDM by Measey et al. (2012); C) Overlap analysis of the maximum entropy SDM and the SDM by Measey et al. (2012), with red highlighting regions with higher probability of the ensemble SDM and blue showing higher probability values for the SDM by Measey et al. (2012). Colour saturation increases with deviation of the models. Areas where both SDMs yield similar probability values are displayed in white. (PDF) [file pone.0154869.s003.pdf]
